# Cognition and emotional decision-making in chronic low back pain: an ERPs study during Iowa gambling task

**DOI:** 10.3389/fpsyg.2014.01350

**Published:** 2014-11-25

**Authors:** Stefano Tamburin, Alice Maier, Sami Schiff, Matteo F. Lauriola, Elisa Di Rosa, Giampietro Zanette, Daniela Mapelli

**Affiliations:** ^1^Section of Neurology, Department of Neurological and Movement Sciences, University of VeronaVerona, Italy; ^2^Department of Medicine, University of PadovaPadova, Italy; ^3^Section of Neurology, Pederzoli Hospital, Peschiera del GardaVerona, Italy; ^4^Department of General Psychology, University of PadovaPadova, Italy; ^5^Human Inspired Technologies Research Center, University of PadovaPadova, Italy

**Keywords:** chronic pain, Iowa gambling task (IGT), decision-making, event-related potentials (ERPs), low back pain

## Abstract

Previous reports documented abnormalities in cognitive functions and decision-making (DM) in patients with chronic pain, but these changes are not consistent across studies. Reasons for these discordant findings might include the presence of confounders, variability in chronic pain conditions, and the use of different cognitive tests. The present study was aimed to add evidence in this field, by exploring the cognitive profile of a specific type of chronic pain, i.e., chronic low back pain (cLBP). Twenty four cLBP patients and 24 healthy controls underwent a neuropsychological battery and we focused on emotional DM abilities by means of Iowa gambling task (IGT). During IGT, behavioral responses and the electroencephalogram (EEG) were recorded in 12 patients and 12 controls. Event-related potentials (ERPs) were averaged oﬄine from EEG epochs locked to the feedback presentation (4000 ms duration, from 2000 ms before to 2000 ms after the feedback onset) separately for wins and losses and the feedback-related negativity (FRN) and P300 peak-to-peak amplitudes were calculated. Among cognitive measures, cLBP patients scored lower than controls in the modified card sorting test (MCST) and the score in this test was significantly influenced by pain duration and intensity. Behavioral IGT results documented worse performance and the absence of a learning process during the test in cLBP patients compared to controls, with no effect of pain characteristics. ERPs findings documented abnormal feedback processing in patients during IGT. cLBP patients showed poor performance in the MCST and the IGT. Abnormal feedback processing may be secondary to impingement of chronic pain in brain areas involved in DM or suggest the presence of a predisposing factor related to pain chronification. These abnormalities might contribute to the impairment in the work and family settings that often cLBP patients report.

## INTRODUCTION

Cognition indicates the brain’s acquisition, processing, storage and retrieval of information, but is also used to describe integrative neuropsychological processes such as mental imaging, problem solving and perception, and is pertinent to emotion and affect (Moriarty et al., 2011).

Among cognitive processes, decision making (DM) is a *complex process that encompasses a range of functions through which motivational processes make contact with action selection mechanisms to express one behavioral output rather than any of the available alternatives* (Rogers, 2011). DM depends on a number of control functions, including selection and inhibition, working memory, planning, emotion, estimation, and other processes included in the domain of the executive functions (EFs). Among these functions, choice evaluation, response selection, and feedback processing play a major role (Fang et al., 2009). Feedback processing is pivotal, in that assigning a positive or negative valence to an option on the basis of previous experience is the prerequisite for the evaluation and anticipation of action outcomes and for an efficient response selection (Mapelli et al., 2014).

The anatomical substrate of DM is a complex network including the prefrontal cortex (PFC), the anterior cingulate cortex (ACC), the fronto-striatal and limbic loops, and some subcortical structures and DM abnormalities are common in patients with lesions or diseases affecting these areas (Gleichgerrcht et al., 2010).

In an attempt to mimic real-life DM scenarios, Bechara et al. (1994) developed the Iowa gambling task (IGT), which simulates, in laboratory environment, DM strategy by factoring the uncertainty of promises and outcomes, as well as reward and punishment. Performance on the IGT is negatively affected by neurological and psychiatric disorders (Brand et al., 2006; Dunn et al., 2006; Mapelli et al., 2014), neurodegenerative changes affecting the PFC (Ernst et al., 2002; Manes et al., 2002; Clark and Manes, 2004; Fellows and Farah, 2005), and deficits in working memory (Manes et al., 2002) and fluid intelligence (Roca et al., 2009).

Longstanding evidence indicate that chronic pain, i.e., pain persisting for 3 months or longer (Merskey and Bogduk, 1994), may have a negative impact on cognition (Moriarty et al., 2011), including working memory, long-term memory and recognition (Grace et al., 1999; Luerding et al., 2008), attention (Grace et al., 1999), EFs, and DM (Weiner et al., 2006; Verdejo-Garcia et al., 2009). Due to its biological salience, pain is an attention-demanding sensory process, but cognitive changes cannot be simply attributed to the attentional demand of ongoing pain.

Morphometric magnetic resonance imaging (MRI) demonstrated gray matter atrophy in the dorsolateral PFC (Apkarian et al., 2004a). Functional MRI showed that, in chronic pain patients, experimental noxious stimuli cause decreased activity in brain regions identified for acute pain (Peyron et al., 2000; Apkarian et al., 2005) and increased activity in regions that are not part of the spinothalamic pathway, mainly the PFC and related subcortical structures (Apkarian et al., 2005). These findings indicate that chronic pain is associated with reduced gain in brain regions involved in acute pain and increased gain in areas outside the classical *pain matrix*. They also suggest that chronic pain may impinge the PFC and the related network and could be considered a cognitive state that may compete with other cognitive abilities, especially those utilizing the PFC, such as DM (Damasio, 1996; Fuster, 2001).

It is important to exercise caution in interpreting these neuropsychological data, because the majority of cognitive abnormalities have been documented in patients with fibromyalgia (Grace et al., 1999; Luerding et al., 2008; Verdejo-Garcia et al., 2009) and cannot be generalized to other chronic pain conditions. Studies in patients with chronic low back pain (cLBP) yielded discordant findings, in that some of them documented reduced attention, visuospatial skills, and cognitive flexibility (Weiner et al., 2006), but the cognitive profile was nearly normal, except slight DM changes, in another report (Apkarian et al., 2004b).

The goal of the present study was to add evidence in this field, by exploring the cognitive profile of a specific type of chronic pain, i.e., cLBP. cLBP patients underwent a neuropsychological battery to explore different cognitive functions and we focused on emotional DM abilities by means of IGT. Abnormalities in different tests would indicate reduced cognitive abilities secondary to the affective and attentional load of pain. At variance, changes in single cognitive functions would favor the hypothesis of specific mechanisms associated with chronic pain. What’s more, focusing on emotional DM might help understanding whether PFC changes documented in neuroimaging studies do translate into cognitive changes.

To explore the cortical correlates of DM, we measured behavioral responses and recorded their neurophysiological cortical correlates with electroencephalogram (EEG) and event-related potentials (ERPs) during IGT in a subgroup of cLBP patients and controls. The monitoring of feedback during DM task evokes a large cortical response mainly localized over central electrodes, which can be separated in a feedback-related negativity (FRN) and a P300, with the former representing an early appraisal of feedback on a binary classification of good vs. bad outcome, and the latter resulting in a later top–down controlled evaluation process that is related to both the valence and the magnitude of the feedback (Gehring and Willoughby, 2002; Yeung and Sanfey, 2004; Hajcak et al., 2006; Holroyd et al., 2006; Wu and Zhou, 2009; Cui et al., 2013; Ferdinand and Kray, 2013; Mapelli et al., 2014).

## MATERIALS AND METHODS

### SUBJECTS

We recruited 24 normal subjects, who volunteered as controls, and 24 patients with cLBP (Merskey and Bogduk, 1994) and pain duration >6 months (**Table 1**), for a total of 48 participants. Baseline demographical conditions (sex, age, education) were not significantly different between patients and controls. All participants gave signed informed consent prior to participation to the study and the protocol had been explained in details to them. The study was approved by the local ethics committee of the Department of Neurological and Movement Sciences, University of Verona.

**Table 1:** Demographic variables in patients and controls.

	cLBP patients (*n* = 24)	Controls (*n* = 24)	*P* value
Age (years)	47.7 ± 9.1, range 35–69	46.1 ± 17.5, range 23–71	0.70^†^
Gender (M/F)	10/14	15/9	0.25^‡^
Education (years)	12.1 ± 4.1, range 5–18	13.5 ± 5.2, range 5–21	0.31^†^


*Continuous variables are expressed as mean ± SD, range. ^†^*P* value from unpaired *t*-test (continuous variables). ^‡^*P* value from the Fisher’s exact test (dichotomous variable). cLBP, chronic low back pain*.

The inclusion/exclusion criteria for patients and controls were: age 18–70, normal or corrected to normal vision, absence of neurological or psychiatric disease, no drugs with psychotropic or neurological effects, mini mental state examination score (MMSE; Folstein et al., 1975) >24.

Chronic low back pain patients had a mean pain duration of 72.9 ± 55.8 months (range: 12–180; median: 24). Average pain intensity was rated before the neuropsychological and IGT evaluation and was 5.1 ± 2.7/10 (range: 2–10; median: 5) on a 0–10 numerical rating scale (NRS). At the time of the evaluation, none of the patients was on chronic treatment, except non steroidal anti-inflammatory drugs when needed, but none of them took any painkiller on the day of testing. The mean score on Beck Depression Inventory (BDI) was 5.0 ± 3.5/39 (range: 1–14; median: 4) which indicated minimal depression, and anxiety score on the State Trait Anxiety Inventory (STAI) Y2 was 45.1 ± 4.9/80 (range: 31–54; median: 46), which indicated mild anxiety.

### COGNITIVE MEASURES

Neuropsychological status was assessed individually by experienced neuropsychologists with a well-validated battery of five tests. The assessment lasted 1 h, with each of the five tests being given to the patients and controls one after the other in the same order. The test list include:

#### Digit span

The digit span test, a subtest of the Wechsler memory scale (Wechsler, 1945), is the format used most often for measuring span of immediate verbal recall and working memory. The test consists of seven (from 2 digits to 8 digits) pairs of random number sequences that the examiner reads aloud at the rate of one a second. The patient’s task is to repeat each sequence exactly as it is given.

#### Modified card sorting test (MCST)

This test is a shorter version (Caffarra et al., 2004) of the Wisconsin card sorting test (Heaton et al., 1993) and assesses the ability to solve problems in response to changing stimuli, the ability to shift and maintain set, and to utilize feedback.

#### Stroop test

This test measures sustained attention and some aspects of EFs, such as the ability to elaborate relevant and irrelevant dimensions in parallel and to inhibit an automatic response while performing a task based on conflicting stimuli (Stroop, 1935; Caffarra et al., 2002).

#### Trail making test (TMT)

This test is divided in parts A and B and evaluates attention, motor speed and EFs (Reitan, 1992).

#### Interference memory task (10 and 30 s)

This test is based on the Brown–Peterson paradigm (Brown, 1958; Peterson and Peterson, 1959) and is a subtest of the neuropsychological battery *esame neuropsicologico breve 2* (short neuropsychological examination version 2; Mondini et al., 2011). This test quantifies the objects that can be held in working memory while preventing participants from using mnemonics or other memory techniques separate from the working memory to increase recall capacity.

### IOWA GAMBLING TASK

Decision-making was assessed with the IGT (Bechara et al., 1994). Even if it was originally designed in analogical mode, in our study the IGT was implemented in a computerized version (Mapelli et al., 2014). The experiment ran with the E-Prime 2 software (Psychology Software Tools, Pittsburgh, PA, USA) installed on a personal computer equipped with a 17-inch monitor.

**FIGURE 1:**
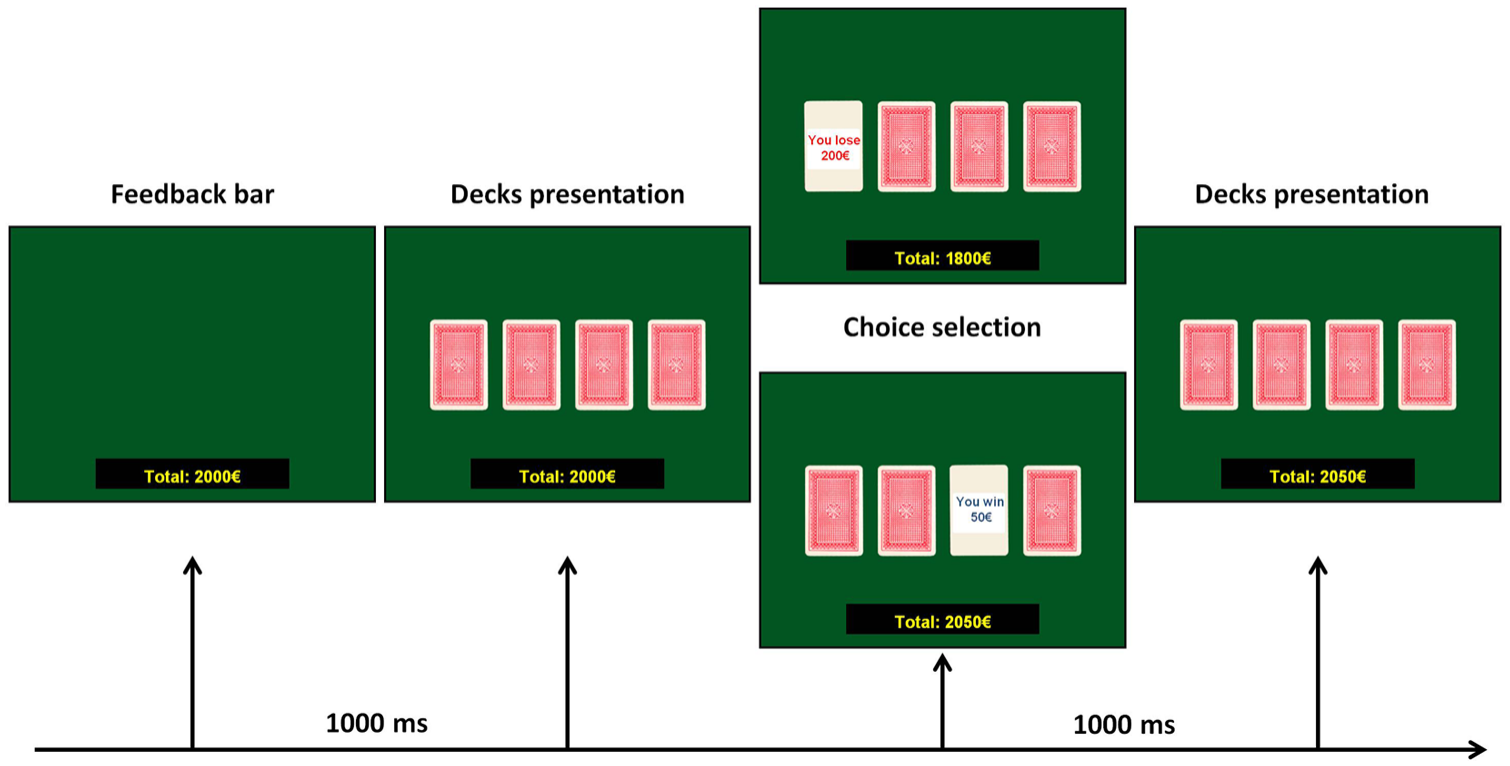
**Experimental flow of the IGT task**.

The task consisted in the presentation, on a computer screen, of four decks named A, B, C, and D. Each card in these decks can bring a win or a loss: participants were requested to gain as more as possible, choosing consecutively one card from any of the four decks, until the task shuts off automatically after 100 cards. The back of each deck looks the same, but decks differ in composition. Decks A and B are considered *disadvantageous*, because they bring big wins but also expensive losses, producing a net loss of 250€ every 10 cards. Decks C and D are considered *advantageous* ones because they bring small wins, but smaller losses, causing a net gain of 250€ every 10 cards. The instructions given to the participants were the following: *in this screen you can see four decks, two of them are advantageous and two are disadvantageous. Each card of these decks can bring a win or a loss: the goal of this task is to win as much money as possible, and avoid losing money as much as possible, starting from a virtual budget of 2000€.* Participants did not know the number of choices and, moreover, which were the advantageous or the disadvantageous decks. Participants saw on the screen the amount of money that they won or loose; this amount was updated after each choice. The experimental flow of the IGT task is shown in **Figure 1**.

The performance in the IGT test was measured using different parameters. The *total amount of money* was the money at the end of the test. The *modal value of deck choices* was explored by calculating the mode of the distribution of the deck choices for each subject of the two groups. The *learning IGT score* was calculated according to previous reports (Bechara et al., 1994; Fukui et al., 2005; Mapelli et al., 2014). To this aim, the 100 picks were divided into five blocks of 20 cards. For each block, the difference between the number of cards picked from *advantageous* decks (C and D) minus those picked from *disadvantageous* ones (A and B) was calculated. In this way, five *learning IGT scores*, one for each block, were obtained for each subject, and the comparison between these scores was considered as an index of learning. An increasing value of the *learning IGT score* from the first to the last block indicates a preference for *advantageous* decks and the learning of the right pick strategy. Finally, the *total IGT score* was calculated by means of the difference between overall *advantageous* choices minus overall *disadvantageous* ones.

### EEG RECORDING

Electroencephalogram and ERPs were recorded in a subgroup of 12 controls and 12 cLBP patients. During the IGT, the EEG was acquired from an array of 32 Ag/AgCl electrodes through a Micromed electrode system. Electrodes were identified by brain hemisphere (odd numbers = left, even numbers = right) and general cortical zone (F = frontal, C = central, T = temporal, P = parietal, and O = occipital) and they were mounted on an elastic cap, according to the International 10–20 system (Oostenveld and Praamstra, 2001). The left and right mastoids served as reference, while the vertical and horizontal eye movements were recorded with two electro-oculogram (EOG) electrodes, placed below and at the outer canthus of the left eye. The ground electrode was located at POz channel. The rating sample was 512 Hz, electrodes impedance were <5 kΩ; a digital band-pass filter (0.1–30 Hz) and notch filter (50 Hz) were applied off-line.

### EVENT-RELATED POTENTIALS

Electroencephalogram data were processed oﬄine using the EEGLAB software (Delorme and Makeig, 2004). Epochs were locked to the feedback presentation (4000 ms duration, from 2000 ms before to 2000 ms after the feedback onset), and the averaging procedure was performed separately for positive and negative feedbacks. Artifact correction was performed using baseline correction in the -500–0 ms time window and independent components analysis technique (Makeig et al., 1996; Delorme and Makeig, 2004).

The FRN amplitude was calculated as the peak-to-peak amplitude difference between the maximal positivity in the 150–250 ms time window and the minimal negativity in the 250–310 ms time window after feedback presentation in the Fz channel because FRN is maximal in the fronto-central midline (Yeung et al., 2005; Hewig et al., 2007; Li et al., 2009).

The P300 amplitude was calculated as the peak-to-peak amplitude difference between the minimal negativity in the 250–310 ms time window and the maximal positivity in the 310–450 ms time window after feedback presentation, from Pz channel because P300 is maximal at the parietal midline (Gehring and Willoughby, 2002; Cui et al., 2013; Mapelli et al., 2014).

### STATISTICAL ANALYSIS

All tests were carried with the IBM SPSS version 20.0 statistical package. For the comparison of baseline demographic conditions (patients vs. controls), the unpaired *t*-test was used for continuous variables and the Fisher’s exact test for dichotomous ones. For continuous cognitive and IGT outcomes, we used the unpaired *t*-test in case of normal distribution, otherwise the non parametric Mann-Whitney *U* test was applied. The dichotomous cognitive variables and the modal distribution of deck choices were explored with the Fisher’s exact test. The correlation between cognitive and IGT measures and clinical variables (depression and anxiety scores, chronic pain intensity, and duration) was analyzed with the Pearson’s coefficient. Learning strategy in the IGT was analyzed with a mixed model repeated-measures ANOVA (within-subjects factor: block, 1 to 5; between-subject factor: group, controls vs. patients) and *post hoc t*-test with Bonferroni’s correction. Homogeneity of variance was analyzed with the Levene’s test. The data were transformed (logarithmic transformation) before submitting them to ANOVA in case of an inequality in the variances. The FRN and P300 amplitudes were submitted to a mixed model repeated-measures ANOVA (within-subjects factor: condition, win vs. loss; between-subject factor: group, controls vs. patients) and *post hoc t*-test with Bonferroni’s correction. Results are reported as mean ± SD except when otherwise specified. *P* < 0.05 (two-tailed) was taken as the significance threshold for all the tests.

## RESULTS

### COGNITIVE MEASURES

Modified card sorting test right categories were significantly lower (*p* = 0.02) and modified card sorting test (MCST) perseverative errors were significantly higher in patients vs. controls (*p* = 0.03), while the other cognitive scores did not significantly differ between the two groups (**Table 2**). The number of MCST right categories was negatively and significantly influenced by the intensity of pain (Pearson’s coefficient = -0.76, *p* = 0.009). The number of perseverative errors was significantly correlated with pain duration (Pearson’s coefficient = 0.79, *p* = 0.007).

**Table 2:** Cognitive measures in patients and controls.

	cLBP patients (*n* = 24)	Controls (*n* = 24)	*P* value
Digit span forward	5.6 ± 0.5	6.1 ± 0.1	0.14
Digit span backward	3.4 ± 0.7	3.8 ± 0.4	0.13
MCST right categories	4.5 ± 1.9	5.8 ± 0.4	0.02*
MCST perseverative errors	4.0 ± 5.6	0.8 ± 1.1	0.03*
Stroop test time	19.2 ± 7.6	14.0 ± 5.8	0.08
Stroop test errors	1.4 ± 1.6	1.1 ± 1.4	0.57
TMT part A	29.7 ± 9.5	25.3 ± 7.3	0.20
TMT part B	92.1 ± 36.8	86.0 ± 23.2	0.59
Interference memory task 10 s	6.9 ± 2.5	8.4 ± 0.5	0.07
Interference memory task 30 s	7.0 ± 1.8	7.8 ± 1.2	0.23

Continuous variables are expressed as mean ± SD, range. *flags significant *p* values when comparing cLBP patients vs. controls. cLBP, chronic low back pain. MCST, modified card sorting test; TMT, trail making test.

### IGT BEHAVIORAL RESULTS

The *total amount of money* at the end of the IGT was lower in cLBP patients (1492 ± 603€) vs. controls (2069 ± 893€; *p* = 0.014). Depression score (BDI), anxiety score (STAI Y2), duration and intensity of pain were not significantly correlated with the *total amount of money*. The *modal value of deck choices* significantly differed between patients and controls, in that 54% of cLBP patients and 83% of controls preferred *advantageous* decks (Fisher’s exact test: *p* = 0.012; **Table 3**).

**Table 3:** The *modal value of deck choices* in patients and controls.

	cLBP patients	Controls	Total
*Advantageous* decks	13	20	33
*Disadvantageous* decks	11	4	15
Total	24	24	48

*Here is reported the type of deck that was the preferred one in cLBP patients and controls (i.e., the mode of the distribution of deck choices). There was a significant difference between the two groups (Fisher’s exact test: *p* = 0.012). cLBP, chronic low back pain*.

**FIGURE 2:**
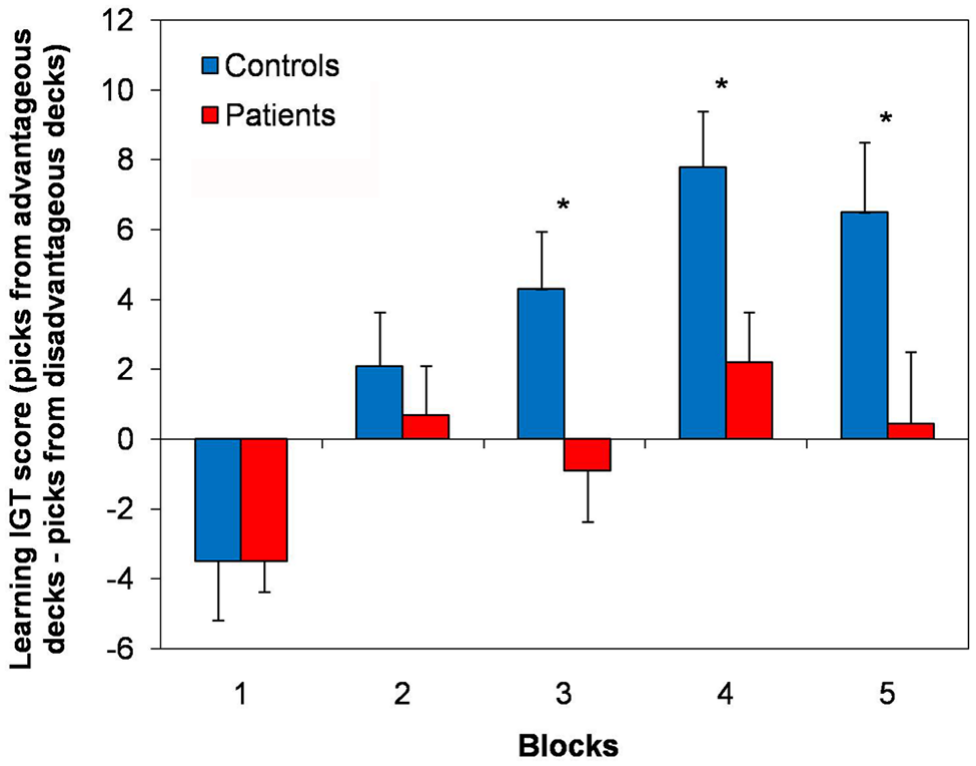
**Learning strategy in the IGT.** Here are shown the *learning IGT scores* across the five different blocks of the IGT in cLBP patients and controls. A learning process was present in controls, in that the *learning IGT score* progressively ameliorated throughout the five blocks. No clear learning strategy was found in cLBP patients, whose *learning IGT score* did not improve across different blocks and fluctuated close to 0. Vertical error bars equal 1 SEM. *p < 0.05 (after Bonferroni’s correction) for cLBP patients vs. controls comparison. cLBP, chronic low back pain; IGT, Iowa gambling task.

When analyzing the distribution of the picks across the experimental blocks, normal controls showed an exploratory strategy, in that at the beginning of the test they explored single blocks and continued picking cards from the same block until they learned whether the deck was *advantageous* or not and, once learned, they preferred the advantageous decks. At variance, the picks of the cLBP patients did not follow a clear strategy, but they seemed to fluctuate randomly across *advantageous* and *disadvantageous* decks. Normal controls showed a learning process during the task, in that the *learning IGT score* progressively ameliorated throughout the five blocks of the test. At variance, no clear learning strategy was found in cLBP patients, whose *learning IGT score* did not improve across different blocks and fluctuated close to 0 (**Figure 2**). Repeated-measures ANOVA showed a main effect of the factors block [*F*(4,184) = 13.01; *p* < 0.001], group [*F*(1,46) = 6.11; *p* = 0.036] and a significant block × group interaction [*F*(4,184) = 2.84; *p* = 0.04] on the *learning IGT score*. *Post hoc* analysis with Bonferroni’s correction showed that the *learning IGT score* was significantly higher in controls vs. patients in blocks 3, 4, and 5 (**Figure 2**). To rule out any possible effect of concomitant depression, patients were divided in those with and without depression according to BDI (cut-off = 5/39) and the between-subjects factor depression was submitted to repeated-measures ANOVA, which documented that neither the factor depression [*F*(1,22) = 0.8; n.s.] nor the block × depression interaction [*F*(1,22) = 1.9; n.s.] significantly influenced the *learning IGT score*.

Depression score (BDI), anxiety score (STAI Y2), duration and intensity of pain were not significantly correlated with the *total IGT score*.

### ERPs RESULTS

The subgroups of cLBP patients (*n* = 12) and controls (*n* = 12) did not significantly differ for age, sex and education. Among cognitive measures, the MCST right categories were significantly lower (cLBP patients: 4.0 ± 2.0, controls: 5.6 ± 2.7; *p* = 0.02) and MCST perseverative errors were significantly higher (cLBP patients: 4.6 ± 4.5, controls: 1.4 ± 1.0; *p* = 0.04) in patients vs. controls, while the other outcomes did not significantly differ between the two groups. For IGT, the *total amount of money* was lower in cLBP patients (1460 ± 692€) vs. controls (2027 ± 571€; *p* = 0.04). Repeated-measures ANOVA showed a main effect of the factors block [*F*(4,88) = 7.32; *p* < 0.001], group [*F*(1,22) = 4.45; *p* = 0.047] and a significant block × group interaction [*F*(4,88) = 2.63; *p* = 0.04] on the *learning IGT score*.

**FIGURE 3:**
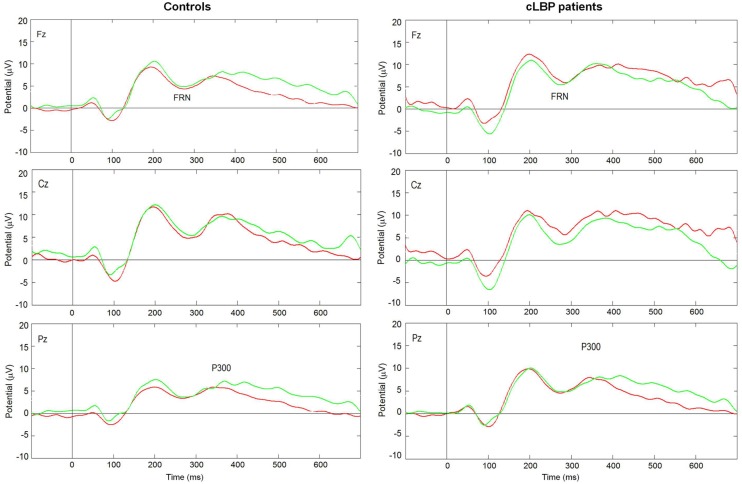
**Grand average ERPs in the Fz, Cz, and Pz channels to wins (green lines) and losses (red lines) in controls and cLBP patients.** cLBP, chronic low back pain; ERPs, event related potentials; FRN, feedback-related negativity.

The grand-average ERPs in patients and controls are displayed in **Figure 3**. There was a prevalence of the number of trials for wins (controls: 73.8 ± 3.8, cLBP patients: 76.1 ± 3.8, n.s.) vs. losses (controls: 17.9 ± 2.3, cLBP patients: 17.0 ± 2.7, n.s.), but this was balanced between the two groups.

**FIGURE 4:**
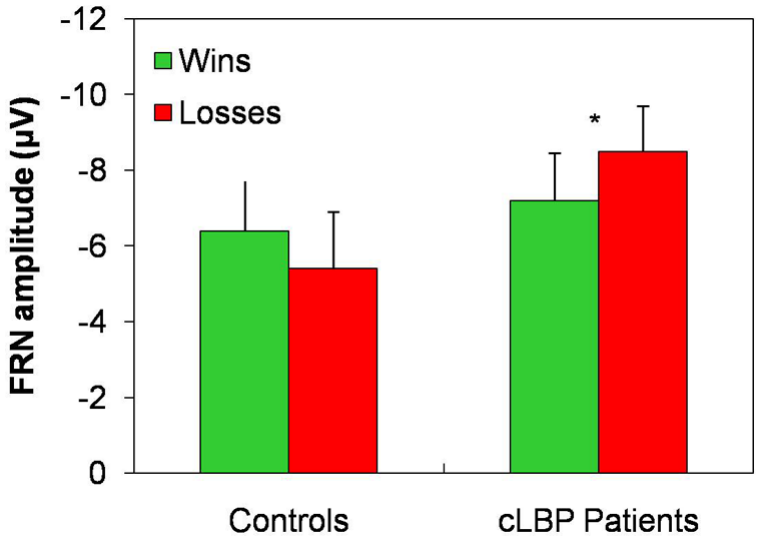
**FRN amplitude in the Fz channel.** Vertical error bars equal 1 SEM. *p < 0.05 (after Bonferroni’s correction) for wins vs. losses comparison. cLBP, chronic low back pain; FRN, feedback-related negativity.

The FRN amplitude in the Fz channel was higher to wins than losses in controls, while the opposite happened in patients (**Figure 4**). Repeated-measures ANOVA showed a significant condition × group interaction [*F*(1,22) = 4.8; *p* = 0.04], while the factors condition [*F*(1,22) = 0.05; n.s.] and group [*F*(1,22) = 1.0; n.s.] did not significantly affect FRN amplitude. *Post hoc* analysis with Bonferroni’s correction showed that the FRN amplitude was significantly higher to losses than wins in patients. The FRN amplitude difference for the two types of feedback (i.e., FRN amplitude to wins – FRN amplitude to losses) was significantly different between the two groups (controls: 1.1 ± 3.2; patients: -1.3 ± 1.9; unpaired *t*-test, *p* = 0.04).

**FIGURE 5:**
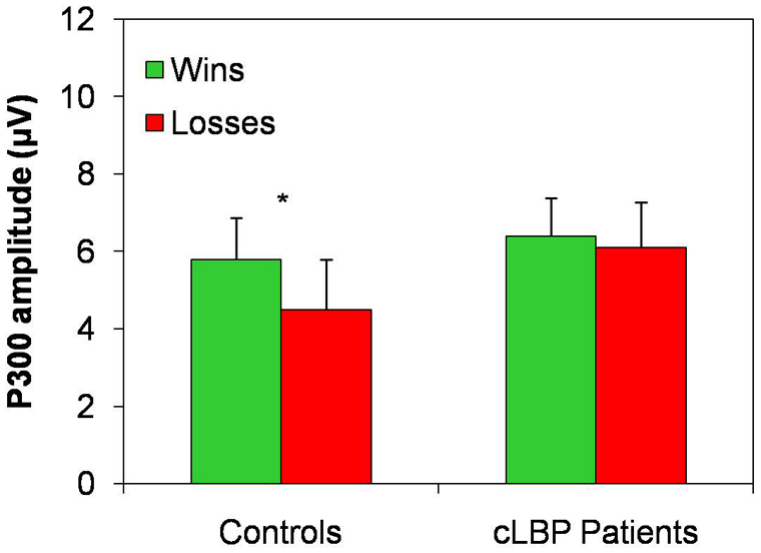
**P300 amplitude in the Pz channel.** Vertical error bars equal 1 SEM.*p < 0.05 (after Bonferroni’s correction) for wins vs. losses comparison. cLBP, chronic low back pain.

The P300 amplitude in the Pz channel was higher to wins than losses in controls, while this difference was absent in patients, being the P300 amplitude similarly high for both types of feedback (**Figure 5**). Repeated-measures ANOVA showed a significant effect of the factor condition [*F*(1,22) = 9.6; *p* = 0.005] and a significant condition × group interaction [*F*(1,22) = 4.7; *p* = 0.04], while the factor group [*F*(1,22) = 0.5; n.s.] did not significantly affect P300 amplitude. *Post hoc* analysis with Bonferroni’s correction showed that the P300 amplitude was significantly higher to positive than negative feedback in controls, while no difference between the two types of feedback was found in patients.

The P300 amplitude difference for the two types of feedback (i.e., P300 amplitude to wins – P300 amplitude to losses) was significantly different between the two groups (controls: 1.3 ± 1.5; patients: 0.2 ± 1.0; unpaired *t*-test, *p* = 0.04).

Feedback-related negativity and P300 amplitude were not influenced by depression score (BDI), anxiety score (STAI Y2), duration and intensity of pain.

## DISCUSSION

In the present study, we explored cognitive functions and DM in cLBP patients and focused on emotional DM abilities by exploring behavioral responses and their neurophysiological correlated during IGT (Bechara et al., 1994). Our data documented that, among cognitive measures, cLBP patients scored lower than controls only in the MCST and that pain duration and intensity were significantly correlated with the degree of impairment in this test. Behavioral IGT results documented worse performance and the absence of a learning process in cLBP patients compared to controls, with no effect of pain characteristics. ERPs findings suggested abnormal feedback processing in patients during IGT.

Previous reports on cognitive functions in chronic pain reported conflicting results, in that abnormalities were not consistent and the tasks explored differed across studies (Moriarty et al., 2011). What’s more, robust cognitive changes were mainly documented in patients with fibromyalgia, a chronic pain condition that is nearly always associated with depression, which may have biased the interpretation of the results. Our findings are in keeping with this bulk of literature, as we found that, out of the large battery of tests, only MCST scores were abnormal in cLBP patients. A previous study documented normal score in Wisconsin card sorting test in cLBP patients, but the very small sample (six patients) might have reduced the power of the statistical analysis (Apkarian et al., 2004b). MCST explores verbal feedback (right, wrong) processing and set shifting. Set shifting appeared to be preserved in our patients because of normal score in trail making test (TMT) part B. We may thus speculate that the abnormalities with MCST resulted from a difficulty in feedback elaboration in the dorsolateral PFC.

We found that the intensity and duration of pain were significantly correlated to MCST scores. Pain duration and intensity were quite variable among our patients and this may represent a bias. However, based on our findings, we may hypothesize that pain might represent a competing task leading to worse and slower functioning of the dorsolateral PFC, which is involved in MCST performance. This view is in keeping with morphological MRI studies, which showed reduced size of the dorsolateral PFC in chronic pain patients (Apkarian et al., 2004a), and that the dorsolateral PFC shrinkage can be reverted by pain treatment suggesting abnormal plasticity to continuous nociceptive afferents (Rodriguez-Raecke et al., 2009, 2013; Seminowicz et al., 2011). It may thus be speculated that intense chronic pain might engage the dorsolateral PFC and cause the abnormalities in MCST, while long pain duration could trigger pathological plastic changes that may be more difficult to revert in patients with long-lasting pain.

Depression and anxiety did not correlate to the MCST performance in our patients, excluding a possible role of these factors. A limitation of the present study is that we did not explore the role of other factors, such as deprivation of social contacts, agility, physical training and life style changes, which together might have also contributed to the MCST abnormalities (Rodriguez-Raecke et al., 2009, 2013).

Iowa gambling task data showed impairment of both the total amount of money and the learning strategy. cLBP patients won significantly less money than controls and their IGT score did not change throughout the blocks indicating the absence of a learning curve during the test. The IGT is a relatively difficult task, but normal controls succeeded in keeping the initial amount of money, while patients lost on average a quarter of the sum. The different outcome in the two groups depended on the presence of a learning strategy in controls, who explored the four decks in the first two blocks of the test, then chose preferentially the advantageous ones. At variance, patients choices appeared largely random ones, and there was a higher number of disadvantageous picks in this group. Depression, anxiety and pain characteristics (i.e., pain intensity and duration) did not influence IGT performance.

To the best of our knowledge, only two studies explored IGT in patients with chronic pain, namely in cLBP and complex regional pain syndrome (Apkarian et al., 2004b) and in chronic migraine (Biagianti et al., 2012). Both these previous reports found that IGT performance were worse in chronic pain patients and that this outcome was not or minimally influenced by depression, anxiety and pain characteristics. Our data differ from those of Apkarian et al. (2004b), in that they found a learning strategy, which was delayed in comparison to controls, in cLBP patients. This difference might be ascribed to our IGT protocol, which was slightly different from the majority of previous studies, in that we told the participants that two of the decks were advantageous and two were disadvantageous (Bechara et al., 2000).

The analysis of feedback-related ERPs offered some insight on the brain mechanisms underlying the bad IGT performance in our patients. To better explore the different stages of feedback processing, we analyzed two ERPs components, namely the FRN and P300.

While FRN was slightly larger for positive vs. negative feedback in normal controls, the opposite happened in our patients, who showed a significantly higher amplitude of this component to losses than wins. The FRN reflects early feedback appraisal on a binary good vs. bad classification, is an index of the violation of the expectations of the subject rather than of the absolute valence of the feedback and is generated in the ACC (Gehring and Willoughby, 2002; Holroyd et al., 2006; Oliveira et al., 2007; Jessup et al., 2010; Alexander and Brown, 2011; Schuermann et al., 2011). Our data suggest that cLBP patients seem to invert the correct placement of feedback according to the good vs. bad outcome basic classification. However, this finding should be interpreted with caution because of the absence of the FRN effect in controls. The reasons for the absence of the FRN effect in our normal subjects might include the relatively old age of some of the controls (Hämmerer et al., 2011; West et al., 2014), the personality profiles and/or genetic variables (Mueller et al., 2014), which were not measured in the present study, or the experimental protocol that differed from some of previous studies, in that the subjects were told that two decks were advantageous and two were disadvantageous.

Controls had a significantly larger P300 to wins than losses, while this component was similarly large to both types of feedback and not significantly different between the two conditions in our patients. The P300 is a more complex phenomenon that reflects the valence of the feedback, contributes to performance monitoring and behavioral adaptation (Schuermann et al., 2011; Cui et al., 2013; Ferdinand and Kray, 2013) and is influenced by attention and working memory updating (Donchin and Coles, 1988; Polich, 2007). The P300 typically shows the *positivity effect* (i.e., a larger amplitude to positive than negative feedback), which is supposed to reflect a positive feedback as more task relevant, because it signals that the intended goal has been achieved (Ferdinand and Kray, 2013). Similar P300 amplitude to both types of feedback in cLBP suggests that patients are unable to differentiate positive and negative outcomes even at this higher-order stage of outcome processing and that they cannot use the information from previous trials and errors for planning future decisions. The abnormally high amplitude of P300 in both conditions might be interpreted as some sort of ceiling effect due to difficulties in tuning the amplitude of this ERPs component in relation to feedback valence.

Behavioral and ERPs abnormalities in cLBP patients might be explained in light of current knowledge of the functional anatomy of DM, which involves a brain network including the amygdala, the ventromedial and the dorsolateral PFC, the ACC, as well as ventral and dorsal striatum (Delazer et al., 2009). IGT and MCST impairment has been documented in many different clinical conditions involving this network, (Bechara et al., 1996, 2000; Rahman et al., 1999, 2006; Fellows and Farah, 2005; Torralva et al., 2009). Healthy aging may also affect the performance in these two tests (Finucane et al., 2002; MacPherson et al., 2002; Kovalchik et al., 2005; Cauffman et al., 2010; Eppinger and Kray, 2011).

Two anatomo-functional hypotheses may be set forth to explain the mechanisms underlying our ERPs findings. Activity in the ventromedial PFC was found to be associated with the fluctuations of pain intensity in cLBP (Baliki et al., 2006; Foss et al., 2006). It may be hypothesized that pain-related activity in the ventromedial PFC might have resulted in an imbalance between ventromedial and dorsolateral PFC leading to the present ERPs abnormalities.

Sensitivity to negative stimuli has been associated with the function of the amygdala (Bechara et al., 1999), which is involved in processing the affective dimension of pain (Giesecke et al., 2005) and influences descending inhibitory pain control through the periaqueductal gray matter (Neugebauer et al., 2004). Based on MRI findings of decreased gray matter bordering the amygdala in patients with cLBP (Ung et al., 2014), we may speculate that continuous nociceptive barrage to the amygdala in patients might cause a dysfunction of this brain structure leading to alteration in feedback processing.

The neuropharmacology of the anatomical network subserving DM points to dopamine (DA) and serotonin. DA is the main neuromodulator of the fronto-striatal loop, and plays a key role (Assadi et al., 2009; Rogers, 2011) in reward processing during reinforcement learning (Schultz, 2002; Frank et al., 2004) and in learning and outcome monitoring (Hämmerer and Eppinger, 2012). Patients with Parkinson’s disease, which is characterized by brain DA reduction and DA manipulation by treatment, show an impairment in DM abilities (Hämmerer and Eppinger, 2012; Mapelli et al., 2014). It may be speculated that changes in DA levels might have blocked the physiological dopaminergic bursts and dips (Frank et al., 2004), which together shape the behavioral responses to positive and negative feedbacks. This view is in keeping with a rodent model, which explored an IGT-like task in rats with pain, and documented that rats performed similarly to our patients and that DA levels were reduced in their ventromedial PFC and amygdala (Pais-Vieira et al., 2009). This model would fit well with the ERPs abnormalities in cLBP patients along with the difficulties in learning a strategy during IGT. Serotonin plays also a relevant role in DM (Gleichgerrcht et al., 2010). Some of our patients showed mild levels of depression, but the absence of any significant effect of depression on IGT findings seems to rule out a possible contribution of the serotoninergic dysfunction.

In contrast to MCST results, IGT abnormalities were not related to any pain variable. We hypothesize that they may represent a predisposing factor for pain chronification and in predicting those patients, who are at risk for developing chronic pain after a futile peripheral tissue damage. Studies on pain chronification have recently shifted from peripheral nerve and spinal cord mechanisms to cortical and limbic phenomena (Baliki et al., 2012). Future prospective studies assessing cognitive functions, including IGT, in patients with acute pain and correlating eventual chronification to their impairment should better explore this hypothesis.

The present IGT abnormalities are similar to those found in pathological gamblers (Goudriaan et al., 2005), as well as in a wider spectrum of neuropsychiatric conditions that share the presence of impulse control disorder and include borderline personality disorder (Schuermann et al., 2011), attention-deficit/hyperactivity disorder and bipolar disorder (Ibanez et al., 2012), and problem gambling (Oberg et al., 2011). Chronic pain patients often have to decide whether to take an analgesic or to change their habits to manage pain. Pain killers have an advantage in the short term (high reward) but, in the long term, they might result in adversive consequences such as side effects or addiction (higher punishment). Otherwise, alternative choices, such as physical activity, cognitive-behavioral therapies or combined treatment (low reward) might result more advantageous in the long term (lower punishment). The IGT impairment in cLBP patients might have an important influence on the selection between various therapeutic options. None of our patients presented symptoms of medication overuse or dependency-like behavior, but exploring IGT changes in patients with drug abuse might be interesting and assessing whether IGT may predict the excessive use of pain killer would have an important role in avoiding this frequent complication of chronic pain.

In conclusion, we documented that cLBP patients show poor performance in DM, as assessed with MCST and IGT. These abnormalities might contribute to the impairment in the work and family settings that often cLBP patients report. Future studies should explore whether these changes may predict the functioning in everyday life.

## Conflict of Interest Statement

The authors declare that the research was conducted in the absence of any commercial or financial relationships that could be construed as a potential conflict of interest.
